# Acute Pediatric Health Risks from Elastomer Thermolysis—PAH Emission Scenarios at School Receptors Following an Industrial Tire Fire

**DOI:** 10.3390/molecules31101659

**Published:** 2026-05-14

**Authors:** Kamil Pająk, Andrzej R. Reindl

**Affiliations:** Department of Environmental Toxicology, Faculty of Health Sciences, Medical University of Gdansk, 80-210 Gdansk, Poland; kamil.pajak@gumed.edu.pl

**Keywords:** pediatric exposure, mutagenic equivalency, ILCR, atmospheric dispersion, PAHs, scrap tire fire

## Abstract

Uncontrolled scrap tire fires represent high-intensity episodic emission events that pose severe toxicological threats to urban environments. This study employs atmospheric dispersion modelling to quantify the impact of a tire stockpile fire on a distal educational receptor, evaluating two distinct dynamic stages of the event: an initial high-intensity open flame scenario (E1, 4 h) and a prolonged smouldering/suppression scenario (E2, 6 h), induced by firefighting interventions. Results reveal extreme pollutant loading at the receptor site during E1, with PM_10_ and SO_2_ concentrations peaking at 23,766 $\frac{\mu g}{m^{3}}$ and 7821 $\frac{\mu g}{m^{3}}$ respectively, indicating an immediate risk of acute respiratory distress. The organic fraction was dominated by volatile organic compounds (VOCs) (8691 $\frac{\mu g}{m^{3}}$) and a ∑16 PAHs flux of 313.9 $\frac{\mu g}{m^{3}}$. Toxicological assessment identified Benzo[a]pyrene (BaP) as the primary driver of health hazards, contributing approximately 70% to the carcinogenic risk profile. A critical disparity was observed between Mutagenic Equivalency (MEQ) of 18.32 and Toxic Equivalency (TEQ) of 15.37, suggesting that standard monitoring significantly underestimates the biological threat to sensitive paediatric populations. These findings demonstrate that acute, oxygen-limited tire combustion creates a concentrated toxic slug of high-molecular-weight PAHs. The study underscores the necessity of integrating mutagenicity-based models into emergency response protocols to accurately safeguard vulnerable communities against the long-term toxicological legacy of elastomer thermolysis.

## 1. Introduction

Uncontrolled industrial waste fires represent a critical environmental challenge, where hazardous atmospheric emissions directly threaten public health and necessitate advanced disaster management strategies. Among the most hazardous episodic events in the industrial landscape are large-scale scrap tire fires, which release complex pyrogenic mixtures leading to acute environmental degradation [1,2]. Unlike chronic industrial emissions, tire fires are characterized by highly inefficient, oxygen-limited combustion of a high-thermal-mass fuel, generating residues that persist in the environment for decades [3,4]. When these events occur proximal to sensitive urban receptors, such as educational institutions, the potential for acute and chronic health degradation becomes a primary concern for toxicologists and risk assessors.

Tire rubber is a sophisticated, engineered chemical matrix, comprising approximately 50% natural and synthetic polymers (primarily styrene-butadiene rubber—SBR), 25% carbon black, and 10–15% metallic reinforcements [3,5,6]. During uncontrolled combustion, particularly in the smouldering conditions characteristic of large stockpiles, this matrix undergoes incomplete thermal degradation and thermolysis. This leads to the thermal synthesis of high-molecular-weight (HMW) polycyclic aromatic hydrocarbons (PAHs), many of which exhibit potent carcinogenic and mutagenic properties [5,7]. The transport of these pollutants to distal school receptors is governed by the chaotic dynamics of the planetary boundary layer (PBL). Large fires generate sufficient buoyancy to penetrate the capping inversion, often leading to the “fumigation” effect, where pollutants are mixed down to the surface, creating toxic hotspots [8]. The vulnerability of the pediatric population at these sites is a critical concern; children exhibit higher ventilation rates relative to their body weight and possess developing biological systems more susceptible to toxic insults [9]. Research has linked PAH exposure to significant health risks [7].

The quantification of this risk requires a high-resolution Incremental Lifetime Cancer Risk (ILCR) framework. While the Toxic Equivalency (TEQ) approach is essential [10,11], it may underestimate the risk of DNA damage. The Mutagenic Equivalency (MEQ) framework reveals a mutagenic hazard, as compounds like Benzo(b)fluoranthene and Indeno[1,2,3-cd]pyrene exhibit higher mutagenic equivalency factors (MEFs) than their corresponding toxic factors [7,12]. Furthermore, firefighting interventions, such as the application of water or foam agent, soften shifts flame combustion into a smouldering phase, surging the emission factors of products of incomplete combustion (PICs) by several orders of magnitude [13,14].

Despite the public health significance, a major gap exists in linking high-resolution emission factors with validated atmospheric dispersion models and in situ multi-pathway risk assessments (ILCR). Standard monitoring restricted to 16 priority PAHs frequently underestimates the total aromatic burden, as forensic characterizations have identified upwards of 165 PAH-related compounds in tire fire soot [15]. To definitively separate fire fallout from chronic traffic abrasion, forensic markers such as the Zinc-Selenium (Zn-Se) correlation and specific PAH isomeric ratios must be employed [16,17].

This study utilizes a holistic “source-to-receptor” analytical framework to reconstruct a major landfill tire fire event. By integrating atmospheric dispersion modelling based on the Hybrid Single-Particle Lagrangian Integrated Trajectory (HYSPLIT) model with demographic-specific health risk models (ILCR), we evaluate the transition from an acute atmospheric crisis to a long-term toxicological legacy at a distal school site. This research provides a robust, evidence-based foundation for public health authorities to safeguard sensitive populations and implement targeted remediation strategies in the aftermath of large-scale industrial disasters.

## 2. Results

### 2.1. Toxicological Burden and Acute Exposure Levels in the School Environment

The atmospheric dispersion modelling conducted for the educational receptor reveals significant pollutant loading directly correlated with the emission duration. As detailed in Table 1, the E1 scenario (representing the initial open flame stage) represents the most acute exposure phase, yielding maximum concentrations approximately 1.5 times higher than those observed in the E2 scenario (representing the smouldering phase induced by fire suppression). The school environment was subjected to extreme levels of criteria pollutants, with PM_10_ reaching 23,765.89 $\frac{\mu g}{m^{3}}$ and SO_2_ peaking at 7821.46 $\frac{\mu g}{m^{3}}$ during E1, which poses a severe risk of acute respiratory distress. The organic fraction was characterized by substantial total VOC concentrations (8690.52 $\frac{\mu g}{m^{3}}$) and a ∑16 PAHs flux of 313.92 $\frac{\mu g}{m^{3}}$, dominated by lighter species such as Naphthalene (130.18 $\frac{\mu g}{m^{3}}$) and Phenanthrene (62.61 $\frac{\mu g}{m^{3}}$). Crucially for health risk assessments, the emission of potent carcinogens like Benzo[a]pyrene (10.75 $\frac{\mu g}{m^{3}}$) and Benzo[k]fluoranthene (11.24 $\frac{\mu g}{m^{3}}$) confirms that the plume delivers a high toxicological and mutagenic burden to the receptor site, even in the extended E2 scenario where Benzo[a]pyrene levels remained elevated at 7.17 $\frac{\mu g}{m^{3}}$.

Maximum air concentrations at the receptor point D1 (school) were lower for Scenario E2 (smouldering, 6 h) than for Scenario E1 (open flame, 4 h) by approximately 33% across all measured species. This pattern reflects the lower instantaneous mass emission rate during the smouldering phase, where reduced combustion temperatures and slower fuel consumption rates limit the per-hour mass output despite higher PAH emission factors per unit fuel burned [18]. However, since the smouldering phase typically lasts longer than the flaming phase, integrated population exposure (concentration × time) may be comparable or even higher for E2, underscoring the importance of considering both peak concentrations and exposure duration in risk assessment.

**Figure 1:**
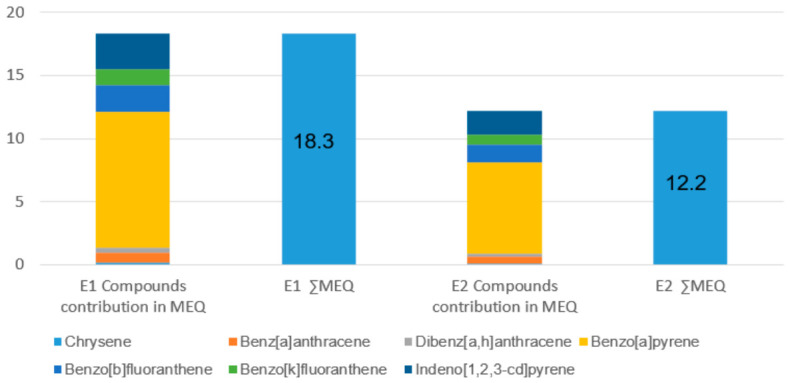
Mutagenic Equivalency (MEQ) concentrations and cumulative ∑MEQ for priority PAHs at the distal school receptor (D1) under fire emission scenarios E1 (high-intensity, open flame stage) and E2 (low-intensity, Smouldering stage).

The mutagenic and toxic equivalency assessments at the distal school receptor (D1) identified BaP as the primary driver of the overall health hazard. In terms of mutagenic potential, BaP accounted for approximately 58.7% of the total burden across both evaluated scenarios (Figure 1). A significant temporal dependency was observed regarding the fire dynamics and emission intensity: the E1 scenario, characterized by a shorter but more intensive combustion duration, resulted in a substantially higher mutagenic air concentration (∑MEQ = 18.32) compared to the more prolonged E2 scenario (∑MEQ = 12.21).

**Figure 2:**
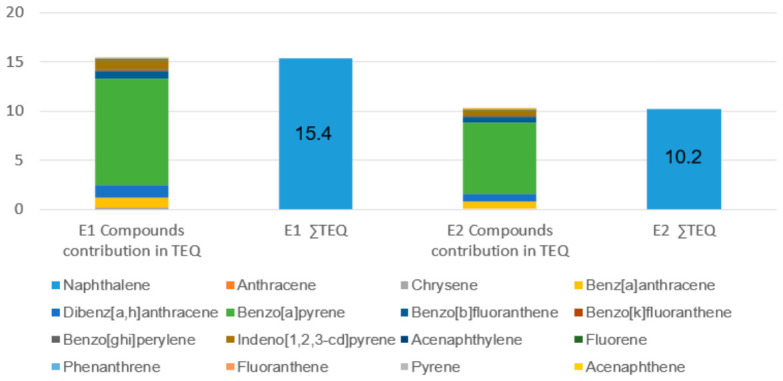
Toxic Equivalency (TEQ) profiles and total ∑TEQ for the 16 priority PAHs at the school receptor (D1), illustrating the impact of varying fire dynamics and emission intensity (E1—Open flame stage vs. E2—Smouldering stage).

Similar trends were observed in the toxicity analysis (Figure 2), where BaP contributed approximately 70% to the total carcinogenic risk profile. The high-intensity E1 scenario generated a total toxic equivalency (∑TEQ) of 15.37, which is roughly 43% higher than that observed in E2. These findings suggest that acute, high-intensity episodic emissions, typical of the early, oxygen-limited phases of scrap tire combustion, create a highly concentrated toxic slug of high-molecular-weight PAHs.

This phenomenon significantly elevates the inhalation dose and associated health risks for vulnerable paediatric populations at school receptors, even when the duration of the emission event is relatively brief.

### 2.2. Acute Exposure and Hazard Indices

The acute risk characterization (Table 2) demonstrated an extraordinary toxicological burden at the pediatric receptor site (D1) during the emission event. Under the high-intensity scenario (E1), the Hazard Quotient (HQ) for PM_10_ reached 528.13, while SO_2_ and NO_x_ exhibited HQs of 11.85 and 3.57, respectively. While the organic fraction, represented by Naphthalene, remained below the acute threshold (HQ < 0.05), the total VOC levels reach HQs of 2.90 (E1) and 1.93 (E2).

## 3. Discussion

### 3.1. Atmospheric Burden and Combustion Dynamics

Uncontrolled fires, especially those involving scrap tires, represent a unique challenge for environmental risk assessment due to the complex chemical matrix of the burning material [24]. As indicated in the current study, tire rubber consists of approximately 50% natural or synthetic rubber, 25% carbon black, 10–15% metallic reinforcements, and smaller fractions of sulphur, zinc oxide, and various additives [6]. Under the oxygen-limited, fuel-rich conditions characteristic of large stockpile fires, the thermolysis of styrene-butadiene rubber (SBR) promotes the de novo pyrosynthesis of a vast spectrum of aromatic hydrocarbons rather than complete oxidation to CO_2_ [5]. Laboratory studies using horizontal reactors have confirmed this mechanism, demonstrating that a decrease in the air ratio significantly increases the yield of volatile and semi-volatile organic compounds [25].

The quantification of emissions in this event confirms a highly inefficient combustion regime, characterized by a substantial release of products of incomplete combustion (PICs). The emission of 1.12 t of CO and 1.34 t of PM_10_ may be considered an indicator of compacted waste tire piles, where high thermal mass creates internal environments with low air-to-fuel ratios [3]. This inefficiency was likely exacerbated by firefighting interventions. The application of water and foams stops flaming combustion but allows the core to continue pyrolyzing at lower temperatures [14]. This transition to a low-temperature smouldering phase may increase emission factors for organic pollutants by several orders of magnitude [14,26]. Such cooling effects prevent complete oxidation, leading to a surge in organic aerosols HMW PAHs, which explains the high relative uncertainty (160.1%) observed in our data [3]. This mechanism is strongly supported by recent findings from Kim et al. (2023) [18] who demonstrated that smouldering emissions inherently possess a higher mutagenicity emission value compared to flaming emissions. This aligns perfectly with our observations regarding the complex PAH profile shift and emphasizes that the prolonged smouldering phase, often induced by firefighting tactics, constitutes a radically elevated mutagenic hazard.

**Figure 3:**
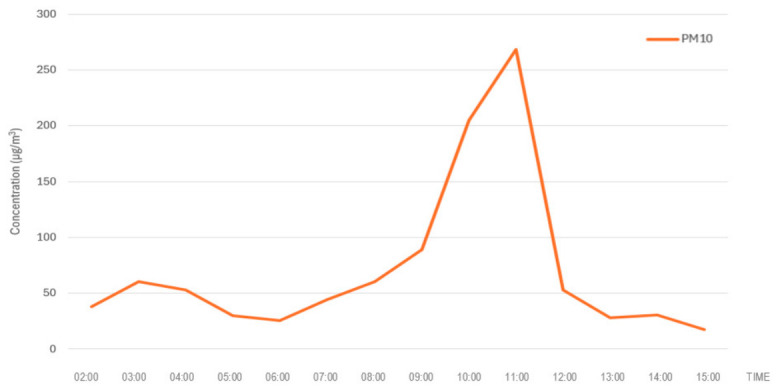
Hourly average ground-level concentrations of PM_10_ recorded at Air Monitoring Station No. 1 (Zalasewo Kornicka) during the tire fire event on 13 October 2025.

**Figure 4:**
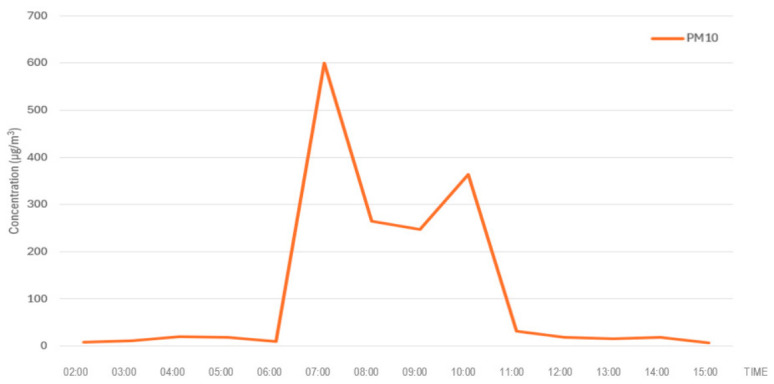
Hourly average ground-level concentrations of PM_10_ recorded at Air Monitoring Station No. 2 (Kruszewnia) during the tire fire event on 13 October 2025.

The dispersion modelling conducted using Operat FB indicated high short-term particulate concentrations in the immediate vicinity of the fire, with PM_10_ reaching 23,765.89 $\frac{\mu g}{m^{3}}$ during the E1 episode in the school environment. Although these values considerably exceed the concentrations recorded at nearby monitoring stations (Figure 3 and Figure 4), such discrepancies are expected due to the sharp spatial gradients characteristic of pyrogenic plumes. The primary factor is the exponential decay of concentration with distance; the school is located in the near-field zone (~850 m), whereas the monitoring stations are situated in the far-field zone (4.0–5.2 km), where significant atmospheric dilution and vertical dispersion occur. Monitoring stations therefore likely captured a diluted fringe of the plume rather than the concentrated core of the near-source exposure. This interpretation is supported by the research of Kumari et al. [27], who reported field-measured PM_10_ concentrations approaching 1.9 × 10^4^ $\frac{\mu g}{m^{3}}$ in the immediate vicinity of uncontrolled tire combustion. The close alignment between our modelled results and these empirical field observations validates the Operat FB output and confirms that rubber combustion can generate exceptionally high particulate loads in near-source environments [28].

Similarly, investigations of uncontrolled landfill tire fires demonstrated substantial emissions of particulate matter and toxic gaseous products associated with incomplete combustion of rubber and synthetic materials [3]. These emissions are typically dominated by soot and particulate-bound toxicants, including polycyclic aromatic hydrocarbons (PAHs) and other organic pollutants. However, evidence from the Grenfell Tower fire further confirms that large structural fires release considerable quantities of soot and particulate debris capable of transporting toxic compounds and producing intense localized environmental contamination, with elevated levels of PAHs, benzene and dioxins detected in soils surrounding the fire site [29]. The atmospheric behaviour of fire-generated particles is strongly size dependent, with particles smaller than 10 μm remaining suspended within the plume and transported over longer distances, while larger particles deposit rapidly near the source, producing pronounced spatial gradients in pollutant concentrations. Consequently, short-term PM levels predicted in the near-field zone of the tire-fire scenario should be considered physically plausible, particularly under relatively low wind speeds that limit plume dilution and favour the accumulation of particulate matter in the immediate downwind area [30].

### 3.2. Toxicological Significance of the PAH Profile

Polycyclic Aromatic Hydrocarbons (PAHs) represent the most hazardous fraction of the emitted plume. Our results demonstrate that while mass emissions were dominated by low-molecular-weight (LMW) compounds—specifically Naphthalene (7.34 kg), Phenanthrene (3.53 kg), and Acenaphthylene (3.10 kg)—the highest toxicological potency is carried by the high-molecular-weight (HMW) species.

The identification of stable forensic markers, such as 13 isomers of MW 302 and 10 isomers of MW 278, confirms the unique pyrogenic fingerprint of tire thermolysis [15]. However, this chemical complexity is inherently underestimated by the standard regulatory monitoring of only 16 priority PAHs. Forensic characterization of high-intensity events, such as the Gatineau tire fire, has identified upwards of 165 PAH-related compounds, including oxygenated, sulphur-, and nitrogen-substituted groups [15]. In this study, the quantitative risk assessment was restricted to the 16 US EPA priority PAHs due to the availability of internationally validated Toxic Equivalency Factors (TEFs). Consequently, the calculated risk levels should be interpreted as a conservative lower-bound estimate of the true toxicological burden. While these 16 compounds serve as reliable surrogate markers for the pyrogenic plume, the exclusion of HMW isomers (e.g., MW 278 and 302) and substituted PAHs—many of which exhibit significant mutagenic potency—suggests that the actual incremental health risk to the pediatric population may be higher than modelled.

The synchronization of modelled plume trajectories with observed concentration spikes at the school facility (Table 1) validates the “source-to-receptor” model. Large-scale tire fires generate sufficient buoyancy to penetrate the planetary boundary layer (PBL), only to be mixed down to the surface via the “fumigation” effect, creating distal hotspots far from the source [8]. At the school receptor, Benzo[a]pyrene (BaP) reached critical levels (10.75 $\frac{\mu g}{m^{3}}$) for determining cumulative toxicity [10,11,31].

Furthermore, the chemical profile of the particulates confirms the tire origin of the incident. The 26-fold increase in Zinc (Zn) serves as a direct chemical fingerprint of the ZnO vulcanization activator used in tire manufacturing [17,32]. To distinguish this event from routine traffic-related abrasion, the strong correlation between Zn and Selenium (Se) provides a unique pyrogenic co-marker profile, typical of high-temperature scrap rubber combustion rather than mechanical wear [16,33]. This multi-elemental and multi-congener approach provides a robust forensic basis for linking the observed pediatric health risk directly to the 9 h “toxic pulse” of the tire fire.

### 3.3. Mutagenic vs. Toxic Equivalency (MEQ and TEQ)

A pivotal finding is the disparity between Toxic Equivalency (TEQ) and Mutagenic Equivalency (MEQ). The absolute values of ∑MEQ (18.32) were significantly higher than ∑TEQ (15.37) for the 4 h scenario. This shift is primarily driven by compounds like Benzo(b)fluoranthene and Indeno[1,2,3-cd]pyrene, which possess higher Mutagenic Equivalency Factors (MEFs of 0.25 and 0.31) than Toxic Equivalency Factors (TEFs) [12].

This “hidden” mutagenic hazard is critical for assessing DNA damage potential in sensitive populations experiencing acute episodic exposure [7]. Mutagenicity assays have previously demonstrated the severe hazard of these events [26]. Crucially, further analysis [7] of the foundational study on tire fire mutagenicity [26] revealed that tire fires exhibit the highest mutagenic emission factor (amount of mutagenicity per kg of fuel burned) among 50 different combustion emissions reviewed. This definitively establishes tire fire emissions as the most harmful combustion emissions ever studied in terms of mutagenicity, reinforcing the hidden genotoxic threat to sensitive populations experiencing acute episodic exposure.

### 3.4. Human Health Risk

A critical point of discussion in this forensic assessment is the interpretation of risk metrics for episodic, high-intensity events. While conventional methodologies typically reserve the Incremental Lifetime Cancer Risk (ILCR) for chronic, decadal exposure scenarios, our findings argue for its utility as a diagnostic measure of the “toxic pulse”—the sudden, disproportionate contribution of a single, high-magnitude event to a child’s cumulative lifetime risk profile.

However, the immediate physiological threat is more precisely captured by the Hazard Quotient (HQ) and Hazard Index (HI) analysis. The fact that the respiratory HI exceeded 500 during the E1 scenario provides a rigorous, non-cancer justification for the acutely hazardous nature of the event. This dual-track approach evaluating both the immediate HQ (acute respiratory threat) and the ILCR (incremental lifetime burden) addresses the inherent methodological complexity of tire fire emissions.

The quantitative health risk assessment revealed that inhalation of tire fire emissions at the school receptor (D1) poses a substantial carcinogenic hazard (Table 3). In all evaluated scenarios, the Total ILCR exceeded the US EPA’s of 10^−6^, with scenario E1 reaching levels above 10^−4^ for the adult demographic, indicating a priority risk level.

A critical temporal dependency was identified: despite the shorter exposure window, the E1 scenario (4 h) yielded ILCR inhalation values approximately 1.5 times higher than the E2 scenario (6 h). This 1.5-fold correlation directly reflects the higher peak concentrations observed in E1, demonstrating that the carcinogenic risk was driven primarily by the intensity of the emission source rather than the duration of the event. This confirms that the high-intensity emission phase following initial ignition creates a toxic slug of concentrated high-molecular-weight PAHs, significantly increasing the acute (inhalation-based) and chronic risk burden for receptors located downwind.

A congener-specific analysis of the risk stacks revealed that Naphthalene was the primary contributor to the inhalation ILCR, accounting for approximately 32% of the total risk loading across all demographics. Other significant contributors included Phenanthrene (15.4%) and Acenaphthylene (13.5%), which reflect the high abundance of low-molecular-weight (LMW) compounds characteristic of styrene-butadiene rubber (SBR) thermolysis. While the absolute ILCR values were numerically higher for adult staff due to longer modelled exposure durations, the risk for school children in scenario E1 (3.78 × 10^−5^) is particularly critical. Children are physiologically more vulnerable due to their higher ventilation rates per kilogram of body weight, ensuring a more rapid intake of contaminants per unit of time compared to adults. These findings highlight that even episodic, short-term exposures during school hours can lead to lifelong toxicological consequences and significant DNA injury in the paediatric population.

Pediatric populations, due to their higher weight-normalized ventilation rates and developing respiratory systems, exhibit a heightened physiological susceptibility to the extreme concentrations of PM_10_ and SO_2_ reported here. The magnitude of the HQ values validates the necessity of immediate evacuation protocols and emergency sheltering, as the 4 h exposure window alone delivered a toxicological dose that would typically be distributed over several years of background ambient exposure.

Furthermore, the synchronization of these findings confirms that the initial phase of tire combustion creates an atmospheric environment that is fundamentally incompatible with public health safety. The extreme HI values suggest that even brief, episodic exposure at this distance from the source (~850 m) poses a critical risk of severe respiratory distress and systemic toxicity. By quantifying both the immediate danger and the lasting “toxic footprint” via ILCR, this study provides a more holistic and realistic framework for assessing the true health cost of industrial disasters in the vicinity of educational receptors.

The calculated Incremental Lifetime Cancer Risk (ILCR) reflects the severe toxicological burden of the event, with values frequently exceeding the acceptable threshold of 10^−6^ [11,34]. While HMW PAHs like BaP dominate carcinogenic risk, LMW species like Naphthalene (7.34 kg) present significant dermal exposure pathways. Naphthalene has been shown to exhibit a high dose absorption efficiency (35.0 ± 5%) when in contact with sweaty skin, suggesting that non-inhalation pathways contribute significantly to the total risk for first responders and nearby residents [9]. The elevated risk at the school site is comparable to the 2016 Seseña tire fire, where cancer risks were 3 to 5 times higher than background levels [35]. Furthermore, the extreme PM_10_ concentrations recorded correlate with acute health risks; epidemiological data suggests that every 10 $\frac{\mu g}{m^{3}}$ increase in 24 h PM_10_ is associated with a 1% rise in daily mortality [36,37]. The presence of SO_2_ (441 kg) further elevates the risk of severe respiratory irritation [13,38]. These findings align with the IARC reclassification of firefighting as a Group 1 carcinogenic occupation, citing PAHs as primary causal agents [39].

While this study focuses on inhalation as the primary exposure vector during the plume passage, it is important to acknowledge the role of dermal absorption, particularly for particle-bound PAHs deposited on the skin during prolonged exposure to the plume [40]. Tire fires are notorious for releasing complex mixtures of HMW PAH species, including dibenzopyrenes and other carcinogenic congeners [15]. However, at the distal school receptor (~850 m from the source), the exposure was characterized by a brief (4–6 h) immersion in the atmospheric plume rather than direct contact with fire-site debris or contaminated ash. Given the acute nature of the event and the physiological vulnerability of the pediatric population to respiratory irritants, inhalation represents the most immediate and critical pathway for systemic toxicant uptake. Nevertheless, post-event deposition of HMW PAHs on school surfaces and soil could represent a secondary, long-term dermal exposure route, which warrants further forensic investigation.

## 4. Study Limitations and Future Perspectives

Although the present study provides a critical evaluation of acute pediatric health risks associated with elastomer thermolysis, several inherent methodological limitations must be acknowledged. Primarily, the atmospheric dispersion modeling utilized a Gaussian framework predicated on steady-state meteorological assumptions for the defined temporal scenarios. This approach inherently simplifies the highly dynamic and turbulent nature of localized pyrogenic plumes, potentially leading to the over- or underestimation of near-field ground-level concentrations in response to micro-meteorological fluctuations. We assumed identical chemical speciation for both burning phases due to the lack of phase-specific data. Furthermore, the estimation of source terms carries intrinsic parameter uncertainties, such as the 160.1% relative uncertainty observed in emission fluxes during smouldering phases, which are dictated by the chaotic mass balance of uncontrolled combustion. Secondarily, the quantitative risk assessment was analytically constrained to the 16 US EPA priority PAHs. Given that forensic characterizations of tire fires indicate the generation of upwards of 165 PAH-related compounds, including highly mutagenic alkylated, oxygenated, and heteroatomic species, the reported Incremental Lifetime Cancer Risk (ILCR) and Mutagenic Equivalency (MEQ) values must be interpreted as conservative, lower-bound estimates. Consequently, the actual biological hazard is likely substantially greater than modeled. Additionally, our ILCR framework explicitly prioritized the inhalation pathway, recognizing it as the most immediate and acute exposure vector during the plume’s passage. However, this focus simplifies the overall cumulative exposure profile. High-molecular-weight PAHs readily partition to particulate matter, subsequently depositing in downwind terrestrial environments. For vulnerable pediatric populations, secondary exposure routes, specifically dermal absorption from contaminated surfaces and the non-dietary ingestion of settled dust via hand-to-mouth behaviours, remain unquantified within this paradigm.

To enhance the representativeness and precision of future forensic environmental assessments, it is imperative to transition toward comprehensive, multi-pathway risk models that integrate the acute atmospheric phase with the long-term depositional legacy, incorporating detailed soil and dust analyses. Methodologically, future investigations should employ non-targeted analytical screening techniques to elucidate the complete mutagenic emission profile beyond the standard priority pollutants. Finally, integrating transient, real-time emission factors derived from large-scale controlled burn experiments into high-resolution, transient dispersion models, such as computational fluid dynamics (CFD), would significantly mitigate existing parameter deviations and refine the temporal accuracy of spatial exposure datasets.

## 5. Materials and Methods

### 5.1. Study Limitations

The study focuses on a large-scale fire at a waste tire storage facility that occurred on 13 October 2025. The ignition was detected at approximately 02:50 a.m., with emergency services notified at 02:55 a.m. The suppression operation, characterized by high-intensity firefighting interventions, lasted 9 h and 27 min. To put out the fire, approximately 60 m^3^ of water and 1300 L of a synthetic foaming agent (Roteor M Premium) were applied. The conflagration affected a surface area of 450 m^2^, with a total combustion volume estimated at 450 m^3^. The rapid application of extinguishing agents during the active phase likely induced a transition from flaming to a smouldering combustion regime, significantly altering the emission profile of products of incomplete combustion (PICs).

### 5.2. Emission Factors and Source-Term Estimation

The total emission flux (E_x_) and the associated uncertainty for specific pollutants (PAHs, PM_10_, VOCs) were quantified following the methodological framework established by Bihałowicz et al. [41] and further refined for tire-specific combustion by Pająk et al. (unpublished data). This approach accounts for the mass balance of the consumed rubber matrix and the variable emission factor (EF) characteristic of uncontrolled scrap tire fires.

### 5.3. Atmospheric Dispersion Modelling and Receptor Concentration

**Figure 5:**
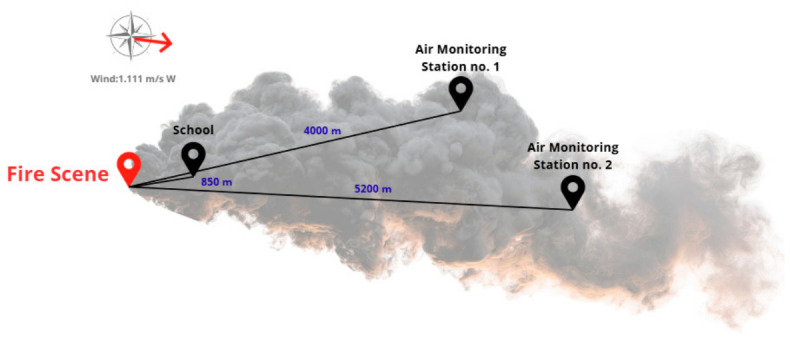
Spatial distribution of the receptor site and regional air monitoring stations relative to the fire source. Red arrow indicate wind direction.

To estimate the ground-level concentrations of pollutants at the receptor point (the educational facility), the Operat FB software (v. 2.9.2) was employed (Figure 5). This model is a standard regulatory tool in Poland for calculating the dispersion of pollutants in the atmosphere, based on the Pasquill–Gifford stability classification and the reference methodology outlined in the Regulation of the Minister of the Environment [42]. The model integrated site-specific meteorological parameters, including wind speed, ambient temperature, and atmospheric stability classes derived from the nearest synoptic station, to calculate the maximum short-term concentrations at the school’s coordinates.

The simulation was conducted for two distinct temporal scenarios to reflect the dynamic nature of the fire and the suppression efforts: scenario E1—a high-intensity emission phase lasting 4 h, representing the initial flaming stage and scenario E2—an extended emission phase of 6 h, accounting for the prolonged smouldering and cooling phase induced by firefighting foam application, where lower temperatures promote the incomplete combustion of high-molecular-weight organic compounds. By employing this dual-scenario framework, the model captures the “toxic pulse” of the flaming phase and the lingering chemical burden of the smouldering phase, providing a more accurate forensic reconstruction of the actual event than a uniform temporal assumption. While an actual fire is a continuous dynamic process, modeling the event minute-by-minute is heavily constrained by the availability of transient emission factors for open-air tire thermolysis. Therefore, establishing two distinct bounding scenarios (E1 representing the open flame stage, and E2 representing the post-intervention smouldering stage) represents the most rigorous approach currently available to capture the macro-dynamics of the fire and the resulting shift in the toxicological profile. This dual-scenario setting ensures the model reflects the actual operational conditions of the incident, enhancing the representativeness and credibility of the health risk outcomes.

The model integrated site-specific meteorological parameters, including wind speed, ambient temperature, and atmospheric stability, to calculate the maximum short-term concentrations at the school’s coordinates (D_1_).

### 5.4. Plume Trajectory and Population Exposure Validation

To complement the Operat FB calculations and evaluate the broader spatial impact, atmospheric transport was simulated using the HYSPLIT (Hybrid Single-Particle Lagrangian Integrated Trajectory) model [43]. Forward trajectories and dispersion fields were computed using the Global Data Assimilation System (GDAS) meteorological datasets with a spatial resolution of 0.5°. The simulation focused on the transport of PM_10_ within the near-surface layer (0–100 m above ground level), representing the primary human breathing zone and accounting for the high concentration of pollutants before significant vertical dilution.

This dual-model approach (Operat FB for local receptor concentration and HYSPLIT for plume trajectory) allowed for the precise identification of the exposed population. By integrating fire-specific source terms, including geographic coordinates, fire duration, and plume rise estimates, into the HYSPLIT framework, we validated that the educational facility (D1) was located directly within the primary transport pathway of the pyrogenic plume throughout the event. This spatial alignment justifies the selection of D1 as a critical receptor for the acute Hazard Quotient (HQ) and Incremental Lifetime Cancer Risk (ILCR) assessment. Detailed HYSPLIT configuration parameters, including the vertical extent of the release and meteorological input files, are provided in the Supplementary Information (S1).

### 5.5. Assessing the Cumulative Chemical Hazard

To characterize the cumulative chemical threat at the educational facility, the toxic equivalency (TEQ) and mutagenic equivalency (MEQ) of the pollutants transported within the smoke plume were determined. These values were derived from the estimated ground-level concentrations at the receptor site, calculated by integrating the source-term emission estimates with the atmospheric dispersion model.

The application of the TEQ framework allowed for the standardization of the complex PAH mixture relative to the carcinogenic potency of benzo(a)pyrene, following the established protocols of Nisbet and LaGoy [44] and the U.S. EPA [11]. Concurrently, the MEQ index was quantified using mutagenic potency factors proposed by Durant et al. [12,45]. This dual approach is essential for assessing pediatric exposure, as specific isomers released during tire thermolysis, such as benzo(b)fluoranthene and indeno[1,2,3-cd]pyrene, often exhibit a higher capacity for DNA damage than their standard toxicological profiles suggest. Consequently, this methodology provided a more comprehensive assessment of the acute genotoxic risk faced by the school population during the fire event.

A comprehensive list of the index values for all 16 priority PAHs used in the calculations is provided in the Supplementary Information (S2).

### 5.6. Health Risk Assessment Framework: Acute and Chronic Perspectives

The health risk assessment was conducted using a dual-track approach to capture both immediate physiological threats and long-term toxicological impacts. This methodology ensures a comprehensive evaluation of the health hazards posed by high-intensity emission episodes, distinguishing between criteria pollutants that cause acute respiratory distress and genotoxic organic fractions that contribute to the cumulative lifetime carcinogenic burden.

The immediate toxicological impact of the plume passage was evaluated using the Hazard Quotient (HQ) for criteria pollutants PM_10_, SO_2_, NO_x_, CO and specific organic fractions (Naphthalene and total VOC) where acute indicators are available. The HQ was calculated using the following formula:

(1) $$HQ=\frac{C_{max}}{RfC_{acute}}$$

where C_max_ is the maximum modeled ground-level concentration at the school receptor [μg/m^3^] and RfC_acute_ is the acute Reference Concentration or Reference Exposure Level (REL) [19,20,21,22,23].

To assess the cumulative effect on the respiratory system, the Hazard Index (HI) was calculated as the sum of HQs for pollutants sharing similar toxicological endpoints (respiratory irritation):

(2) HI=∑HQ

To evaluate the oncogenic hazard posed by polycyclic aromatic hydrocarbons (PAHs) deposited via atmospheric fallout and fire-related particulates, a quantitative risk assessment was performed. The computational framework was based on the Incremental Lifetime Cancer Risk (ILCR) model, in accordance with the U.S. EPA Human Health Evaluation Manual [46]. This approach integrates toxicological potency with site-specific exposure variables, as validated in previous forensic environmental investigations [12,15,44,45,47,48].

While the ILCR model is conventionally associated with chronic, decadal exposure, it was selected here as the most appropriate tool to evaluate the “toxic pulse” of the organic plume. For the broader suite of PAHs (e.g., Benzo[a]pyrene, Chrysene, Dibenz[a,h]anthracene), an acute HQ cannot be determined due to the lack of officially recognized acute RfC_acute_ or RELs in international toxicological databases (e.g., OEHHA, US EPA IRIS). Consequently, the ILCR framework allows for the quantification of this discrete, high-magnitude exposure as an incremental addition to the cumulative lifetime carcinogenic burden of the pediatric population. This dual approach ensures that the hazard posed by the high-molecular-weight PAH fraction is not ignored simply due to the absence of acute-specific indicators, providing a holistic evaluation of both immediate physiological threats and long-term health security.

### 5.7. Target Groups and Exposure Routes

The risk characterization was stratified into two distinct demographic cohorts to account for age-dependent physiological and behavioural differences (1) paediatric population (children aged 1–6 years)—characterized by higher metabolic rates and specific hand-to-mouth activity and (2) adult population (aged 7–70 years)—representing long-term chronic exposure over a typical lifetime.

The study primarily focused on the inhalation pathway, encompassing the intake of both gaseous-phase volatile fractions and resuspendable particulate matter (PM_10_) within the school’s breathing zone during the passage of the pyrogenic plume.

### 5.8. Mathematical Risk Quantification

The ILCR for the inhalation route was calculated by integrating the concentration of contaminants (Cs) with specific exposure factors and the Cancer Slope Factor (CSF). The CSF values were adjusted for body-weight scaling and age-specific ventilation rates to ensure toxicological accuracy. The calculation protocol followed the multidimensional models established by Yang et al. [49] and Deelaman et al. [47], allowing for the estimation of the excess probability of developing cancer as a result of the fire-derived PAH exposure.

## 6. Conclusions

The investigation into the uncontrolled combustion of scrap tires reveals a critical intersection between complex elastomer thermolysis and acute public health risk. Based on the source-to-receptor forensic modeling, the key conclusions are:

− Acute Particulate Exposure: The interaction of high-thermal-mass fuel with oxygen-starved conditions generates a persistent “toxic slug” of high-molecular-weight pollutants, driving PM_10_ concentrations to an extreme 23,765.89 ug/m^3^ at the distal school receptor.
− Underestimation of Biological Hazard: Standard toxicological assessments significantly underestimate the biological threat. The modeled Mutagenic Equivalency (MEQ) exceeded the Toxic Equivalency (TEQ) by a considerable margin, driven primarily by potent isomers of Benzo[b]fluoranthene and Indeno[1,2,3-cd]pyrene.
− Forensic Pyrogenic Signatures: The identification of MW 302 and 278 PAH isomers, alongside the unique Zn-Se correlation, provides a robust framework for distinguishing acute tire fire fallout from chronic urban traffic emissions.
− Severe Carcinogenic Burden: Plume buoyancy coupled with the atmospheric “fumigation” effect delivers critical concentrations of Benzo[a]pyrene (10.75 ug/m^3^ to distal communities, elevating the Incremental Lifetime Cancer Risk (ILCR) to the 10^−4^ priority threshold.

Ultimately, this research necessitates a paradigm shift in disaster management. As our modeled exposure data demonstrate severe toxicological loading during the prolonged event, and the established combustion literature highlights the role of rapid cooling in surging products of incomplete combustion, there is a clear need for tactical firefighting to adapt strategies to minimize prolonged smouldering. Concurrently, public health protocols must transition to mutagenicity-based risk models to safeguard vulnerable populations from the long-term toxicological legacy of industrial tire fires.

## Figures and Tables

**Table 1:** Maximum ground-level concentrations [µg/m^3^] at the school receptor (Point D1) for Scenario E1 (4 h) and Scenario E2 (6 h).

Compounds	Maximum Concentration in Air—Point D1 School $\left[\frac{\mu g}{m^{3}}\right]$	
	Scenario E1 (4 h Emission) Open Flame Stage	Scenario E2 (6 h Emission) Smouldering Stage
CO_2_	806,976	537,983
CO	19,864	13,242
NO_x_	1676	1117
SO_2_	7821	5214
PM_10_	23,766	15,843
CH_4_	4469	2979
**VOC (total)**	**8691**	**5793**
Naphthalene	130.2	86.79
Anthracene	11.10	7.402
Chrysene	12.29	8.19
Benz[a]anthracene	9.70	6.47
Dibenz[a,h]anthracene	1.26	0.84
Benzo[a]pyrene	10.75	7.17
Benzo[b]fluoranthene	8.51	5.68
Benzo[k]fluoranthene	11.24	7.50
Benzo[ghi]perylene	6.91	4.61
Indeno[1,2,3-cd]pyrene	9.15	6.10
Acenaphthylene	54.98	36.65
Fluorene	12.58	8.38
Phenanthrene	62.61	41.74
Fluoranthene	34.76	23.18
Pyrene	30.51	20.34
Acenaphthene	0.78	0.52
**∑_16_ PAHs**	**313.92**	**209.28**

**Table 2:** Acute toxicological risk characterization (HQ and HI) at the pediatric receptor site (D1).

Compounds	HQ		RfC/REL *	
	Scenario E1 Open Flame Stage	Scenario E2 Smouldering Stage	$\left[\frac{\mu g}{m^{3}}\right]$	Reference
CO_2_	0.09	0.06	9,000,000	[19]
CO	0.86	0.58	23,000	[20]
NO_x_	3.57	2.38	470	[20]
SO_2_	11.85	7.90	660	[20]
PM_10_	528.13	352.09	45	[21]
VOC total	2.90	1.93	3000	[22]
Naphthalene	0.04	0.03	3144	[23]
**∑ HI**	**547.44**	**364.96**		

* Reference Exposure Level.

**Table 3:** Calculated Incremental Lifetime Cancer Risk (ILCR) for adults and children via the inhalation pathway at the school receptor (D1).

Compounds	Scenario E1 (4 h) Open Flame Stage		Scenario E2 (6 h) Smouldering Stage	
	Adults	Children	Adults	Children
Naphthalene	2.885 × 10^−5^	1.208 × 10^−5^	1.923 × 10^−5^	0.805 × 10^−5^
Anthracene	0.246 × 10^−5^	0.103 × 10^−5^	0.164 × 10^−5^	0.069 × 10^−5^
Chrysene	0.272 × 10^−5^	0.114 × 10^−5^	0.182 × 10^−5^	0.076 × 10^−5^
Benz[a]anthracene	0.215 × 10^−5^	0.090 × 10^−5^	0.143 × 10^−5^	0.060 × 10^−5^
Dibenz[a,h]anthracene	0.028 × 10^−5^	0.012 × 10^−5^	0.019 × 10^−5^	0.008 × 10^−5^
Benzo[a]pyrene	0.238 × 10^−5^	0.100 × 10^−5^	0.159 × 10^−5^	0.066 × 10^−5^
Benzo[b]fluoranthene	0.189 × 10^−5^	0.079 × 10^−5^	0.126 × 10^−5^	0.053 × 10^−5^
Benzo[k]fluoranthene	0.249 × 10^−5^	0.104 × 10^−5^	0.166 × 10^−5^	0.070 × 10^−5^
Benzo[ghi]perylene	0.153 × 10^−5^	0.064 × 10^−5^	0.102 × 10^−5^	0.043 × 10^−5^
Indeno[1,2,3-cd]pyrene	0.203 × 10^−5^	0.085 × 10^−5^	0.135 × 10^−5^	0.057 × 10^−5^
Acenaphthylene	1.218 × 10^−5^	0.510 × 10^−5^	0.812 × 10^−5^	0.340 × 10^−5^
Fluorene	0.279 × 10^−5^	0.117 × 10^−5^	0.186 × 10^−5^	0.078 × 10^−5^
Phenanthrene	1.387 × 10^−5^	0.581 × 10^−5^	0.925 × 10^−5^	0.387 × 10^−5^
Fluoranthene	0.770 × 10^−5^	0.322 × 10^−5^	0.514 × 10^−5^	0.215 × 10^−5^
Pyrene	0.676 × 10^−5^	0.283 × 10^−5^	0.451 × 10^−5^	0.189 × 10^−5^
Acenaphthene	0.017 × 10^−5^	0.007 × 10^−5^	0.011 × 10^−5^	0.005 × 10^−5^
**∑ ILCR estimated**	**9.026** × **10**^−5^	**3.778** × **10**^−5^	**6.017** × **10**^−5^	**2.519** × **10**^−5^

## Data Availability

The original contributions presented in this study are included in the article. Further inquiries can be directed to the corresponding author.

## Supplementary Materials

The following supporting information can be downloaded at: https://www.mdpi.com/article/10.3390/molecules31101659/s1, Table S1. Fire Source Term and Operational Parameters for Dispersion Modelling. Table S2. Toxic Equivalency Factors (TEF) and Mutagenic Equivalency Factors (MEF) for the 16 priority PAHs used in health risk assessment.
