# Supplementary material for: Acute Pediatric Health Risks from Elastomer Thermolysis—PAH Emission Scenarios at School Receptors Following an Industrial Tire Fire
Source: Molecules. 2026 May 14;31(10):1659. doi: 10.3390/molecules31101659 (PMC13209325; doi:10.3390/molecules31101659)
Supplement: Supplementary file 1 [file molecules-31-01659-s001.zip › molecules-4325811-supplementary.pdf]

S1

Table S1. Fire Source Term and Operational Parameters for Dispersion Modelling

**Parameter**	**Unit**	**Value**	**Source / Note**
Date of event	-	October 13, 2025	Fire Service Logs (SWD)
Time of outbreak	-	02:50 a.m.	Emergency notification at 02:55
Total duration	min	567 (9 h 27 min)	Total suppression time
Geographic coordinates	DD	52.3889 N, 17.0215 E	Source location
Affected area	m^2	450	Estimated fire ground area
Fuel volume (V)	m^3	450	Estimated combustion volume
Fuel type	-	Waste tires	High-density rubber scrap
Release height	m	1.0	Near-ground emission source
Suppression agent: Water	m^3	60	Total volume applied
Suppression agent: Foam	L	1300	Roteor M Premium (Synthetic)

Section S2 Emission Estimation Methodology

Total emissions (Ex) for each pollutant were estimated following the methodological framework proposed by Bihałowicz et al. (2021). The total mass of the emitted substance was calculated using the following equation:

$$EM_x^i = q_y \cdot V_i \cdot EF_{\{x,y\}} \qquad (1)$$

where q_y denotes the bulk density of the waste tires (kg/m^3) accounting for interstitial air voids, V is the estimated volume of consumed fuel (m^3), and $EF_{x,y}$ is the substance-specific emission factor (g/kg). Due to the heterogeneous nature of tire landfill fires, characterized by oxygen-limited regimes and the suppressive effects of water/foam, emissions were temporally averaged assuming a quasi-steady combustion phase.

Note on Temporal Distribution:
For the dispersion modelling (Operat FB), the total calculated mass (Ex) was distributed across two scenarios: E1 (initial 4-hour flaming phase) and E2 (subsequent 6-hour smouldering phase). This distribution accounts for the non-linear emission rates observed during the application of 1300 L of firefighting foam, which altered the combustion temperature and oxygen availability.

S2

Table S2. Toxic Equivalency Factors (TEF) and Mutagenic Equivalency Factors (MEF) for the 16 priority PAHs used in health risk assessment

PAH Compound	TEF[a]	MEF[b]
Naftalen	0,001	-
Antracen	0,01	-
Chryzen	0,001	0,017
Benzo(a)antracen	0,1	0,082
Dibenzo(a,h)antracen	1	0,29
Benzo(a)piren	1	1
Benzo(b)fluoranten	0,1	0,25
Benzo(k)fluoranten	0,01	0,11
Benzo(g,h,i)perylen	0,01	-
Indeno(1,2,3-cd)piren	0,1	0,31
Acenaphthylene	0,001	-
Fluorene	0,001	-
Phenanthrene	0,001	-
Fluoranthene	0,001	-
Pyrene	0,001	-
Acenaphthene	0,001	-

[a] *TEF values according to Nisbet and LaGoy (1992) and U.S. EPA guidelines.*
[b] *MEF values according to Durant et al. (1996, 1999).*
Note: "-" indicates that no validated mutagenic equivalency factor was available or applied for the specific congener.
